# Supplementary material for: In-silico modelling of the mitogen-activated protein kinase (MAPK) pathway in colorectal cancer: mutations and targeted therapy
Source: Front Syst Biol. 2023 Aug 23;3:1207898. doi: 10.3389/fsysb.2023.1207898 (PMC12342002; doi:10.3389/fsysb.2023.1207898)
Supplement: Supplementary file 1 [file Presentation1.pdf]

Supplementary Material

Appendix S1. Model for DBF and TMT

To model the action of DBF we considered the CR-CRN that takes into account the GoF mutation of *KRAS* and we added the reversible reaction (Sommariva et al., 2021b)

$$\mathrm{DBF} + \mathrm{B\text{-}Raf} \underset{k_{dr}}{\overset{k_{df}}{\rightleftarrows}} \mathrm{DBF\_B\text{-}Raf}$$

where DBF_B-Raf is the inactive drug–target complex. According to this model, DBF is expected to behave as an inhibitor of B-Raf activation (Hamis et al., 2021; Morkel et al., 2015) and thus to compete with K-Ras_GTP, that binds the same site of B-Raf. The values of the rates are taken from Hamis et al. (2021) as $k_{df} = 0.106 \cdot 10^{-3}$ nM^{-1}s^{-1}, $k_{dr} = 0.593 \cdot 10^{-4}$s^{-1}. Notice that they are different from those considered by Sommariva et al. (2021b). In the search for the steady state under the action of the drug, the initial values of the protein concentrations have been set equal to the equilibrium values of the network subject to the GoF mutation of *KRAS*. Taking inspiration both from Hamis et al. (2021) and from Sommariva et al. (2021b), the initial values of the drug concentration have been extracted from the interval $[0, 100]$ nM.

In a second set of experiments we also modelled the degradation of DBF by adding to the CR-CRN the following reaction

$$\mathrm{DBF} \xrightarrow{k_{deg}} \emptyset$$

where $\emptyset$ is the so-called zero complex (Feinberg, 1987), and $k_{deg} = 5.79 \cdot 10^{-6}$ s^{-1} (Anderson et al., 2019).

The action of TMT has been described by adding to the mutated network the reactions

$$\mathrm{TMT} + \mathrm{p\text{-}p\text{-}MEK} \underset{k_b}{\overset{k_f}{\rightleftarrows}} \mathrm{TMT\_p\text{-}p\text{-}MEK},$$

$$\mathrm{TMT} + \mathrm{ERK\_p\text{-}p\text{-}MEK} \underset{k_b}{\overset{k_f}{\rightleftarrows}} \mathrm{TMT\_ERK\_p\text{-}p\text{-}MEK},$$

$$\mathrm{ERK} + \mathrm{TMT\_p\text{-}p\text{-}MEK} \underset{k_{84}}{\overset{k_{83}}{\rightleftarrows}} \mathrm{TMT\_ERK\_p\text{-}p\text{-}MEK}.$$

Therefore TMT is expected to behave as an allosteric inhibitor of p-p-MEK (Hamis et al., 2021; Morkel et al., 2015), which in turn acts within the CR-CRN as an enzyme for the double phosphorylation of the substrate ERK. Indeed, in the CR-CRN, ERK is activated by the reaction with p-p-MEK which binds at a certain site, while TMT binds at a different site of the p-p-MEK molecule. Thus the complex TMT_p-p-MEK may bind ERK to form the inert complex TMT_p-p-MEK_ERK, which cannot produce a phosphorylated ERK. The values of the rate constants k_f, k_b have been taken from (Hamis et al., 2021) as $k_f = 0.106 \cdot 10^{-3}$ nM^{-1}s^{-1}, $k_b = 0.12296 \cdot 10^{-2}$ s^{-1}, whereas k_{83} and k_{84} have been taken from (Sommariva et al., 2021a) as $k_{83} = 0.1 \cdot 10^{-1}$ n M^{-1}s^{-1} and $k_{84} = 0.33 \cdot 10^{-2}$s^{-1}. Following (Hamis et al., 2021), the values of the initial concentration of TMT have been chosen in the interval $[0, 2000]$ nM.

From a computational point of view, the models for DBF and TMT are included in the CR-CRN through the function `f_add_drug_Raf_from_file.m` (`f_add_drug_Raf_from_file_deg.m` if also drug degradation must be included in the model) that reads reactions and species to be added from dedicated files. All these functions and files are available at the GitHub repository `https://github.com/theMIDAgroup/CRC_CRN`.

Appendix S2. Model for the GoF of *KRAS*

As thoroughly described in previous works (Sommariva et al., 2021a,b) a mutation resulting in a GoF of protein K-Ras is modelled by acting on the reactions of the network responsible for its deactivation. Within the CR-CRN the reactions involved in switching the active GTP-bound, K-Ras_GTP, into the inactive GDP-bound, K-Ras_GDP are 28 (the complete list of reactions forming the CR-CRN can be found in the supplementary material by Sommariva et al. (2021b)). Namely the reaction

$$\text{K-Ras\_GTP} \xrightarrow{\mathbf{k_{44}}} \text{K-Ras\_GDP}$$

plus 3 groups of the form

$$\mathcal{P}\text{\_GAP} + \text{K-Ras\_GTP} \underset{k^{\mathcal{P}}_{1r}}{\overset{\mathbf{k^{\mathcal{P}}_{1f}}}{\rightleftarrows}} \mathcal{P}\text{\_GAP\_K-Ras\_GTP} \xrightarrow{\mathbf{k^{\mathcal{P}}_{2}}} \mathcal{P}\text{\_GAP} + \text{K-Ras\_GDP}$$

$$\mathcal{P}\text{\_G\_GABP\_GAP} + \text{K-Ras\_GTP} \underset{k^{\mathcal{P}}_{3r}}{\overset{\mathbf{k^{\mathcal{P}}_{3f}}}{\rightleftarrows}} \mathcal{P}\text{\_G\_GABP\_GAP\_K-Ras\_GTP} \xrightarrow{\mathbf{k^{\mathcal{P}}_{4}}} \mathcal{P}\text{\_G\_GABP\_GAP} + \text{K-Ras\_GDP}$$

$$\mathcal{P}\text{\_ShP\_G\_GABP\_GAP} + \text{K-Ras\_GTP} \underset{k^{\mathcal{P}}_{5r}}{\overset{\mathbf{k^{\mathcal{P}}_{5f}}}{\rightleftarrows}} \mathcal{P}\text{\_ShP\_G\_GABP\_GAP\_K-Ras\_GTP} \xrightarrow{\mathbf{k^{\mathcal{P}}_{6}}} \mathcal{P}\text{\_ShP\_G\_GABP\_GAP} + \text{K-Ras\_GDP}$$

where $\mathcal{P}$ is a protein among RP, ERBP, and ERB3P. To model a mutation resulting in a GoF of K-Ras we acted on the reaction rates depicted in bold. Specifically, we simulated three levels of GoF by multiplying the original values of all the considered rate constants (shown in the second column of Table S1) by a scaling factor, namely 0.6, 0.3 and 0. The resulting mutated values of the rate constants are reported in Table S1, column three, four and five, respectively. The values of the scaling factor have been heuristically chosen as an illustrative example of how the proposed model could be possibly tuned to represent different mutations of the same gene. Future effort will be devoted to model actual biologically relevant mutations, such as $\text{KRAS}^{\text{G12C}}$ and $\text{KRAS}^{\text{Q61H}}$ mutation.

From a computational point of view, all the mutations considered in this work have been included within the CR-CRN through the dedicated function `f_define_mutated_condition.m` available at the GitHub repository `https://github.com/theMIDAgroup/CRC_CRN`.

Table S1. Original and mutated values of the rate constants modified to model three mutations resulting in different levels of GoF of the molecule K-Ras.

**Rate constants**	**Original values**	**GoF 60%**	**GoF 30%**	**GoF 0%**
k_{44}	10^{-5}	$6 \cdot 10^{-6}$	$3 \cdot 10^{-6}$	0
$k_{1f}^{\mathcal{P}}$	0.01	0.006	0.003	0
$k_{2}^{\mathcal{P}}$	1.494	0.8964	0.4482	0
$k_{3f}^{\mathcal{P}}$	0.6225	0.3735	0.1867	0
$k_{4}^{\mathcal{P}}$	1.494	0.8964	0.4482	0
$k_{5f}^{\mathcal{P}}$	0.6225	0.3735	0.1867	0
$k_{6}^{\mathcal{P}}$	1.494	0.8964	0.4482	0

REFERENCES

Anderson, M. W., Moss, J. J., Szalai, R., and Lane, J. D. (2019). Mathematical modeling highlights the complex role of AKT in TRAIL-induced apoptosis of colorectal carcinoma cells. *IScience* 12, 182–193

Feinberg, M. (1987). Chemical reaction network structure and the stability of complex isothermal reactors—I. The deficiency zero and deficiency one theorems. *Chem Engin Sci* 42, 2229–2268

Hamis, S. J., Kapelyukh, Y., McLaren, A., Henderson, C. J., Roland Wolf, C., and Chaplain, M. A. (2021). Quantifying erk activity in response to inhibition of the brafv600e-mek-erk cascade using mathematical modelling. *British Journal of Cancer* 125, 1552–1560

Morkel, M., Riemer, P., Bläker, H., and Sers, C. (2015). Similar but different: distinct roles for kras and braf oncogenes in colorectal cancer development and therapy resistance. *Oncotarget* 6, 20785

Sommariva, S., Caviglia, G., and Piana, M. (2021a). Gain and loss of function mutations in biological chemical reaction networks: a mathematical model with application to colorectal cancer cells. *Journal of Mathematical Biology* 82, 55

Sommariva, S., Caviglia, G., Ravera, S., Frassoni, F., Benvenuto, F., Tortolina, L., et al. (2021b). Computational quantification of global effects induced by mutations and drugs in signaling networks of colorectal cancer cells. *Scientific reports* 11, 19602
